# Development and evaluation of *Sargassum* polysaccharides–algal oil nanoemulsion for intranasal treatment of allergic rhinitis

**DOI:** 10.1186/s40643-026-01043-2

**Published:** 2026-03-27

**Authors:** Shi-Yu Hung, Hsiang-Wen Chan, Hao-Hsiang Ku, Chung-Hsiung Huang

**Affiliations:** 1 Department of Food Science, National Taiwan Ocean University, Keelung, 202301 Taiwan; 2 Institute of Food Safety and Risk Management, National Taiwan Ocean University, Keelung, 20224 Taiwan; 3 Center for Marine Bioscience and Biotechnology, National Taiwan Ocean University, Keelung, 20224 Taiwan

**Keywords:** Algal oil, Allergic rhinitis, Intranasal delivery, Nanoemulsion, *Sargassum* polysaccharides

## Abstract

**Abstract:**

Allergic rhinitis (AR) is a prevalent inflammatory disorder of the upper respiratory tract, affecting 20–40% of the global population and severely impairing quality of life. Given the limitations and adverse effects associated with conventional pharmacotherapy, naturally derived bioactives with low toxicity are gaining prominence as alternative interventions. In this study, we developed a bioresource-based nanoemulsion (NE) by integrating *Sargassum* polysaccharides (SP) into algal oil (AO) to enhance intranasal delivery and therapeutic efficacy against AR. Structural analysis confirmed that SP comprised sulfated polysaccharides enriched in fucose, glucose, and galactose. The optimized SP–AO NE, formulated with Tween 80 and prepared via ultrasonic emulsification, exhibited uniform spherical droplets (53.4 ± 1.8 nm), a low polydispersity index (0.3 ± 0.1), and a negative zeta potential (− 29.1 ± 2.8 mV), indicating high colloidal stability and effective oxidative protection of AO during refrigerated storage. In an ovalbumin-induced AR mouse model, intranasal administration of SP–AO NE significantly alleviated nasal rubbing, epithelial hypertrophy, goblet cell hyperplasia, mast cell infiltration, and pulmonary inflammation. Intranasal SP–AO NE treatment decreased IgE levels in serum, nasal lavage, and bronchoalveolar lavage fluids, while enhancing mucosal IgA. In addition, SP–AO NE downregulated IL-4 and TNF-α expression and upregulated TGF-β1, demonstrating a robust immunomodulatory effect. Overall, this work presents a stable, biocompatible, and functional NE that improves intranasal delivery of algal bioactives, offering a promising natural therapeutic strategy for the management of AR.

**Graphical abstract:**

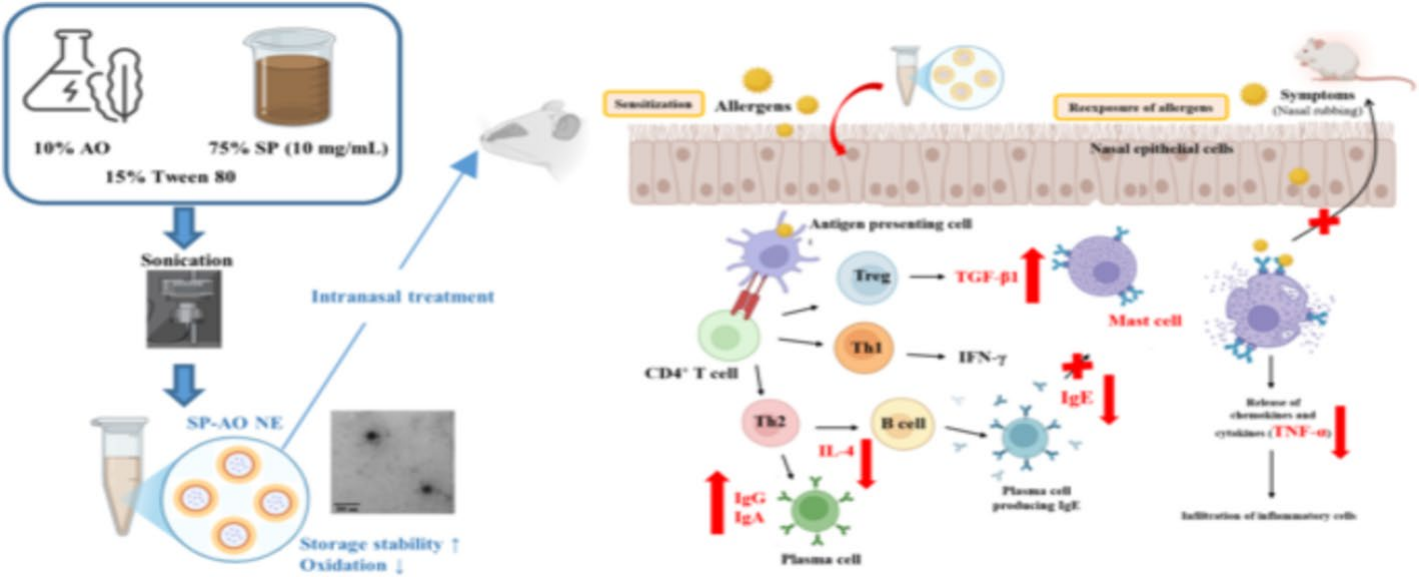

## Introduction

Accounting for 20–40% of cases globally, allergic rhinitis (AR) manifests as a persistent inflammatory disease targeting the upper portions of the respiratory system. Characteristic features include nasal obstruction, watery discharge, pruritus, and sneezing, which occur as a consequence of an excessive immune reaction and immunoglobulin E (IgE) production to environmental allergens (Meng et al. 2020; Bousquet et al. 2020). The immunopathogenesis of AR involves a disrupted balance between T helper (Th)1 and Th2 cells, with Th2 predominance driving the disease. Upon allergen exposure, interaction with antigen-presenting cells drives the differentiation of naïve T cells into Th2 subsets, which secrete interleukin (IL)-4 and other cytokines that promote IgE synthesis and mast cell activation, leading to allergic inflammation (Nur Husna et al. 2022). Additionally, AR patients often exhibit reduced regulatory T (Treg) cell-associated cytokines (e.g. transforming growth factor (TGF)-β1 and IL-10), which further weakens immune tolerance and sustains inflammation (Sun et al. 2016). Th2-derived cytokines also impair epithelial barrier integrity and induce goblet cell hyperplasia, contributing to mucus overproduction (Chegini et al. 2023). Current AR management strategies include allergen avoidance, antihistamines, intranasal corticosteroids, and allergen immunotherapy. While these interventions can alleviate symptoms, their limitations include sedation and cognitive impairment from antihistamines, mucosal damage or systemic side effects from corticosteroids, and the prolonged treatment duration required for immunotherapy (Platt 2014; Min 2010). These drawbacks have encouraged the exploration of natural, low-toxicity alternatives with immunomodulatory potential.

Algal bioactives have attracted considerable attention in this context. Brown seaweeds of the genus *Sargassum* are rich in polysaccharides such as fucoidan and alginate, which exhibit antioxidant, anti-inflammatory, and immune-regulatory activities. *Sargassum* polysaccharides (SP) have been reported to suppress allergic responses by lowering IgE, IL-4, and tumor necrosis factor (TNF)-α levels while enhancing interferon (IFN)-γ and TGF-β1 expression (Chen et al. 2023). Similarly, algal oil (AO), recognized for its high content of the omega-3 lipid docosahexaenoic acid (DHA), has demonstrated immunoregulatory effects by reducing allergic inflammation and promoting Treg differentiation (Han et al. 2015). Compared with fish oil, AO represents a more sustainable and vegetarian-friendly alternative without compromising efficacy.

To improve the bioavailability and mucosal delivery of bioactive components, nanoemulsion (NE)-based systems have emerged as a promising platform. NE, typically characterized by droplet sizes in the nanometer range, enhance the solubility, permeability, and stability of both hydrophilic and lipophilic compounds. They can improve oxidative protection, prolong retention time, and enable controlled or targeted release (Sharma et al. 2024; Preeti et al. 2023). Intranasal administration offers distinct advantages for upper airway diseases such as AR, providing localized therapeutic effects, rapid onset of action, and avoidance of hepatic first-pass metabolism (Watts et al. 2019; Ghori et al. 2015).

In this study, we developed a novel NE system that co-delivers SP and AO for intranasal management of AR in a murine model. Although SP and AO individually exhibit anti-inflammatory and immunomodulatory activities, their combined incorporation into a single nanoformulation has not yet been explored. This integrated delivery strategy is designed to enhance therapeutic outcomes by synergistically regulating the Th2/Treg immune axis. Accordingly, the present work introduces a biocompatible NE platform that couples algal-derived bioactives with an advanced intranasal delivery approach, offering a promising natural intervention for the effective control of AR.

## Materials and methods

### Materials

Unless stated differently, all reagents and chemicals were supplied by Sigma-Aldrich (St. Louis, MO, USA) and Panreac (Castellar del Vallès, Barcelona, Spain), Bionovas Biotechnology (Kingsdale Ave, Toronto, Canada), ScyTek Laboratories (Logan, UT, USA), Difco (Franklin Lakes, NJ, USA), Corning Inc. (Woodland, CA, USA), Genestar Biotechnology (Kaohsiung, Taiwan), Bio SB (Santa Barbara, CA, USA), Vector Laboratories (Newark, CA, USA). Algal oil was purchased from Bio Island (New South Wales, Australia). Fresh *S. fusiforme* was kindly provided by Professor Jui-Sheng Chang (National Taiwan Ocean University, Keelung, Taiwan).

### Preparation and characterization of SP

**Fig. 1:**
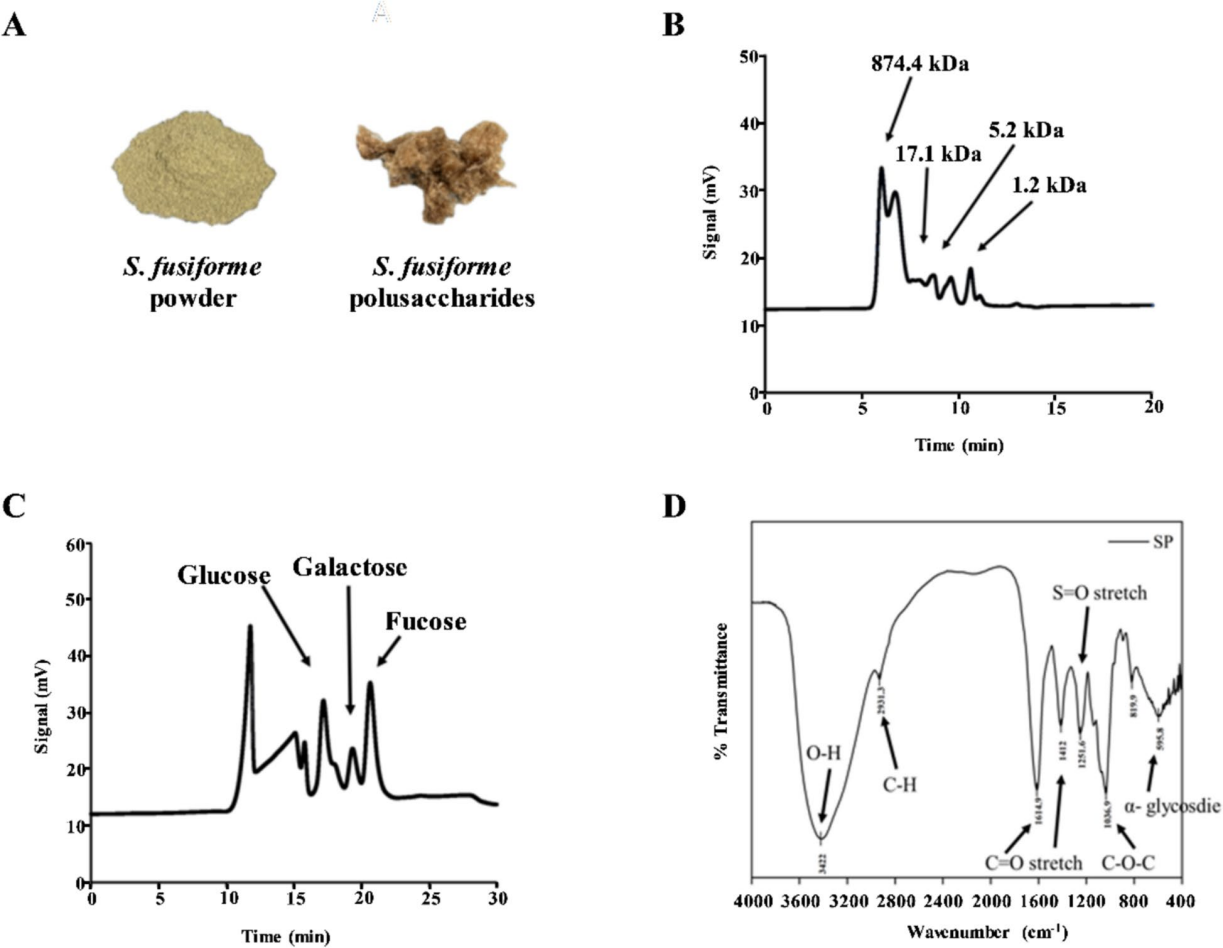
Characterization of SP. **A** Visual appearance of *S. fusiforme* powder and SP. **B** Size-exclusion chromatography (SB-804 HQ) profiles showing the molecular mass distribution of SP. **C** HPLC chromatograms indicating the monosaccharide composition of SP. **D** FTIR spectra revealing the functional groups present in SP

*S. fusiform*e was thoroughly washed in distilled water to eliminate surface epiphytes and salts, and subsequently freeze-dried for 72 h. The dried *S. fusiform*e was milled to a powdered form and screened through a 0.42 mm sieve before storage in a desiccator (Fig. 1A). The powder was made into a solution with 2.5 g per 100 mL of deionized water (w/v) and extracted at 121 °C for 20 min with continuous stirring until cooled. The centrifugation process was conducted at 12,500 × *g* for 20 min, allowing the supernatant to be harvested. Polysaccharides were precipitated by mixing the supernatant with six volumes of 95% ethanol and incubating for 24 h, with subsequent centrifugation performed at 12,500 × *g* for 20 min. The precipitate was freeze-dried for 24 h to acquire crude polysaccharides powder. The crude polysaccharides were solubilized in deionized water at 1% (w/v), desalinated via electrodialysis (Micro Acilyzer S3, ASTOM Corporation, Tokyo, Japan), and fractionated by a 10 kDa molecular mass cutoff ultrafiltration membrane. The retentate (> 10 kDa) was freeze-dried for 72 h to yield purified SP (Fig. 1A), which were preserved in a desiccator prior to use (Chen et al. 2025).

SP (25 mg) was processed by hydrolysis in 2 M trifluoroacetic acid subjected to a temperature of 100 °C over a period of 4 h. After reaching ambient temperature, the mixture was vacuum-concentrated to neutral pH (pH = 7) and reconstituted with 2 mL deionized water. The composition of monosaccharides was determined via high-performance liquid chromatography (HPLC) adopting a Benson Polymeric™ BP-100Pb column (7.8 × 300 mm) maintained at 90 °C. Deionized water served as the mobile phase, delivered at 0.4 mL/min. A refractive index detector coupled with the EC2000 data processing system was employed for detection. Standard monosaccharides including glucose, galactose, fucose, mannose, rhamnose, fructose, mannitol, and sorbitol (0.625–10 mg/mL) were solubilized in deionized water and subsequently filtered through a 0.22 µm membrane, and analyzed to generate calibration curves based on peak area (Liu et al. 2025).

SP was prepared as 10 mg/mL aqueous solutions, filtered (0.22 µm), and degassed by ultrasonication for 30 min prior to HPLC analysis. Molecular mass distribution of polysaccharides was analyzed using a Shodex Asahipak SB-804 HQ column (7.5 × 300 mm) at 60 °C. The mobile phase was prepared with deionized water with 0.02% sodium azide as an antimicrobial agent, flowing at 1 mL/min. Detection was carried out by refractive index detector and data processed using EC2000. A pullulan standard was used to generate a molecular mass calibration curve (Chen et al. 2025).

For Fourier-transform infrared spectroscopy (FTIR) analysis, a mixture was prepared by combining 1 mg of SP with 99 mg of KBr through grinding, dried at 60 °C for 8 h, with the mixture subsequently compacted into pellets under vacuum using a pellet press. The FTIR measurements were conducted utilizing a Thermo Scientific Nicolet iS5 infrared spectrometer (Thermo Fisher Scientific Inc., Waltham, MA, USA) with 16 scans at 8 cm⁻^1^ resolution over the range 4000–400 cm⁻^1^. Pure potassium bromide pellets were used for background correction to identify characteristic functional groups in the SP (Chen et al. 2025).

The SP contained 65% total sugar and 27% reducing sugar (Table 1), as determined using established colorimetric methods (Dubois et al. 1956; Miller 1959). The sulfate content was 14% (Table 1), measured according to the method of the previous study (Dodgson and Price 1962). Endotoxin contamination was assessed using an Endotoxin ELISA Kit (Abbexa Ltd., Cambridge, UK), and endotoxin levels were below 0.01 EU/mL. Batch-to-batch reproducibility was evaluated across triplicate experiments, which showed only limited variation in the contents of total sugar, reducing sugar and sulfate (Table 1). Although the general chemical composition of *Sargassum* can vary with geographic factors such as growth location, harvest season, climate, and water quality (Diharmi et al. 2023), the results demonstrate good reproducibility of the SP preparation process when the same batch of *Sargassum* was used.

**Table 1:** Contents of total sugar, reducing sugar, and sulfate in SP

	% (w/w)
Total sugar	65 ± 0
Reducing sugar	27 ± 1
Sulfate content	14 ± 1

Each value is mean ± standard deviation (n = 3)

### Preparation, characterization and storage stability of NE

A nonionic surfactant, Tween 80, notable for its high hydrophilic–lipophilic balance, was selected as the emulsifier due to its excellent capability to stabilize NE. The optimized formulation consisted of 10% (v/v) AO, 15% (v/v) Tween 80, and 75% (v/v) aqueous phase, as this composition enabled the successful preparation of stable NE with the lowest surfactant content and the highest AO and SP incorporation. The oil phase (10% AO) was first mixed thoroughly with Tween 80 using a vortex mixer. Subsequently, 75% phosphate-buffered saline (PBS) containing SP (10 mg/mL) or PBS alone (for control NE) was slowly added under continuous vortexing to obtain a pre-emulsion. Subsequently, the mixture underwent ultrasonic emulsification using an ultrasonic processor (W-380 sonicator Ultrasonic Processor, 20 kHz converter probe, Heat Systems, Anchorage, AK, USA) operating at 20 kHz for 20 min to produce fine NE (Liu et al. 2025). Dynamic light scattering (DLS) analysis on a Zetasizer Nano ZS (Malvern Instruments, UK) was conducted to characterize the droplet size distribution, polydispersity index (PDI), and surface charge of the NE. For morphological observation, NE was diluted 100-fold in deionized water, and 10 μL was deposited onto a carbon-coated copper grid for 30 min adsorption. Residual liquid was carefully wicked away with filter paper, and the process was repeated three times. Following vacuum drying, the samples on the grids were examined with a transmission electron microscope (TEM; Hitachi HT 7700, Tokyo, Japan) to visualize droplet shape and dispersion. For stability evaluation, samples were held at 4, 25, and 37 °C for a period of eight weeks. At two-week intervals, fixed volumes of NE were withdrawn and analyzed by DLS. To monitor lipid oxidation during storage, NE stored at 4 °C in the dark was assessed weekly using the thiobarbituric acid reactive substances (TBARS) assay. Briefly, NE (0.25 mL) was reacted with thiobarbituric acid (0.5 mL) at 95 °C for 15 min, cooled, and centrifuged (1000 × *g*, 10 min). Supernatant absorbance at 532 nm was measured, and TBARS values were calculated as malondialdehyde equivalents using a 1,1,3,3-tetraethoxypropane calibration curve.

### Murine model of AR

Female BALB/c mice, aged four weeks, were procured from the National Center for BioModels (Taipei, Taiwan). Under controlled conditions (24 ± 1 °C, 40–60% relative humidity, 12 h light/dark cycle), mice were allowed free access to standard chow and water. After allowing the animals to acclimate for one week, experimental procedures were performed in line with institutional standards and received approval from the Institutional Animal Care and Use Committee of National Taiwan Ocean University (NTOU-IACUC-112018M1). A total of five groups were established by random allocation, with six mice assigned to each group (n = 6), including naïve control (NA), ovalbumin (OVA)-induced allergic rhinitis (AR), AR induction and SP treatment (AR-SP), AR induction and AO treatment (AR-AO), and AR induction and SP-AO NE treatment (AR-NE). Sensitization was performed on days 1, 8, and 15 via intraperitoneal injection of 0.2 mL of a solution comprising 0.25 mg/mL OVA and 5 mg/mL Al(OH)₃. A daily intranasal challenge with 20 μL of 40 mg/mL OVA was performed on the mice during days 21–27, and the NA group received PBS. One hour before each challenge, treatment groups received intranasal administration (30 μL/mouse) of their respective formulations. The AO dose corresponded to 10 mg/kg DHA and the SP dose to 1.875 mg/kg. An equal amount of PBS was administered to mice in the NA group. In accordance with the 3R principles, the employed dose was selected based on preliminary in vitro screening, using the highest concentration that did not significantly reduce primary murine cell viability to minimize animal use while maintaining biological relevance. On day 28, mice were euthanized by CO₂ inhalation. The evaluation of allergic manifestations was conducted 10 min post-final challenge with the frequency of nasal rubbing used as an indicator of symptom severity (Wang et al. 2024; Zhang et al. 2020). Before euthanasia, serum samples were collected. After euthanasia, the spleen was aseptically excised and placed in RPMI-1640 medium for splenocyte preparation. Three washes with 0.5 mL PBS were performed in the nasal cavity to collect nasal lavage fluid (NALF). Three consecutive lung washes using 0.5 mL PBS via tracheal cannulation were performed to obtain bronchoalveolar lavage fluid (BALF). All lavage samples were kept on ice and centrifuged before analysis. Nasal and lung tissues underwent fixation in 10% neutral-buffered formalin, paraffin embedding, sectioning, and hematoxylin–eosin (H & E) staining for histopathology, periodic acid–Schiff (PAS) staining for goblet cell hyperplasia, and toluidine blue staining for mast cell identification. Light microscopy (Nikon ECLIPSE TS100, Japan) at 400 × and 1000 × was used to examine stained sections, and ImageJ software (v1.53t, NIH) facilitated quantitative image analysis. The animal experiments were repeated twice independently to demonstrate replicability. Nasal rubbing and histological assessments were performed by experimenters blinded to group assignments.

### Cytokine and antibody determination

Fresh spleens were aseptically collected from euthanized mice and rinsed 3 times with 5 mL sterile RPMI-1640 medium. The spleen tissues were passed through a cell strainer to obtain suspensions of individual cells. After centrifugation at 1200 × *g* for 5 min, the supernatant was discarded. The cell pellets were treated with 0.5 mL RBC lysis buffer for 90 s, followed by quenching with 10 volumes of RPMI-1640 containing 10% fetal bovine serum. For cell viability assessment, splenocytes (6 × 10^6^ cells/mL) were seeded in 96-well plates (100 µL/well) and co-incubated with 1% (v/v) OVA for 48 h. Cells were incubated with 50 µL 3-(4,5- dimethylthiazol-2-yl)-2,5-diphenyltetrazolium bromide (MTT, 1 mg/mL) for 4 h, followed by 100 µL dimethyl sulfoxide, and absorbance was read at 570 nm (Quant, Biotek, Winooski, VT, USA).

For cytokine measurement, supernatants from cultured splenocytes were collected, and the levels of IFN-γ, IL-4, TNF-α, and TGF-β₁ were determined using commercially available enzyme-linked immunosorbent assay (ELISA) kits (Invitrogen, Thermo Fisher Scientific, Waltham, MA, USA) according to the manufacturer’s guidelines. Additionally, serum, NALF, and BALF were assessed for total and OVA-specific immunoglobulins (IgE and IgA) using ELISA kits (Invitrogen, Thermo Fisher Scientific, Waltham, MA, USA) following the recommended protocols.

### Immunohistochemical (IHC) staining

Paraffin Sects. (5 μm) were treated with xylene twice for 5 min each and then rehydrated stepwise through ethanol gradients from 100 to 60%, 5 min per concentration. After triple PBS washes of 5 min each, antigen retrieval was performed through boiling in citrate buffer. Sections were then treated with 6% H₂O₂ for 15 min to quench endogenous peroxidase, followed by blocked with 2.5% goat serum for 1 h. Anti-IL-4 antibody were applied overnight at 4 °C. After washing, sections were incubated with poly-HRP antibody for 1 h. DAB substrate was used for 10 min for color development, followed by hematoxylin counterstaining. Slides underwent a 10 min wash under a gentle flow of tap water, air-dried in the dark, mounted, and observed by a light microscope at 1000 × magnification (ECLIPSE TS100, Nikon, Japan).

### Reverse transcription quantitative polymerase chain reaction (RT-qPCR)

Using TRIzol™ reagent, total RNA was obtained from 50 mg of nasal and lung tissues. Tissues were homogenized at 6000 × *g* for 30 s in three cycles with 1 mL TRIzol™, incubated for 5 min, then added with 0.2 mL chloroform, shaken, and centrifuged at 12,000 × *g* for 15 min. After transferring the aqueous layer to a new tube, RNA was precipitated with 0.5 mL isopropanol for 10 min, centrifuged at 12,000 × *g* for 10 min, washed in 1 mL of 75% ethanol, and centrifuged again at 7500 × *g* for 5 min to collect the RNA pellet. To synthesize cDNA, 5 μg of total RNA was processed with the Maxima H Minus First Strand cDNA Synthesis Kit using oligo(dT)18 together with random hexamer primers. Reverse transcription consisted of a 10-min incubation at 25 °C, a 15 min extension at 50 °C, and a final termination step at 85 °C for 5 min. Quantitative real-time PCR was carried out in 20 μL reactions composed of 10 ng cDNA and 500 nM primers using PowerUp™ SYBR™ Green Master Mix on the ABI QS1 platform (Applied Biosystems, Foster City, CA, USA). The relative expression of IL-4 mRNA, using β-actin as an internal control, was calculated according to the 2^−ΔΔCT^ method. The sequences of primers employed for IL-4 were 5′-TACCAGGAGCCATATCCACGGATG-3′ (forward) and 5′-TGTGGTGTTCTTCGTTGCTGTGAG-3′ (reverse). For β-actin, forward 5′-GTTGGAGCAAACATCCCCCA-3′ and reverse 5′-CGCGACCATCCTCCTCTTAG-3′ primers were used (Huang et al. 2025).

### Statistical analysis

One-way analysis of variance (ANOVA) was used to assess differences between groups, performed with SigmaPlot version 14.0 (Systat Software Inc., San Jose, CA, USA). In cases where ANOVA demonstrated statistical significance, Fisher’s LSD test was applied for post hoc comparisons within a predefined set of group contrasts. Differences were regarded as significant when *p* values were less than 0.05.

## Results

### Characterization SP and NE

The molecular mass distribution of SP was analyzed by HPLC, revealing four major peaks with retention times of 6.1, 8.7, 9.6, and 10.6 min. Corresponding molecular masses were estimated as 874.4, 17.1, 5.2, and 1.2 kDa, respectively, indicating that the extracted SP is a heterogeneous mixture ranging from 1.2 to 874.4 kDa (Fig. 1B). Monosaccharide composition of SP, determined by HPLC following 2 M trifluoroacetic acid hydrolysis, identified three main sugars, including fucose (52.4%), glucose (24.6%), and galactose (23.1%) (Fig. 1C). Characteristic functional groups were verified through FTIR spectral analysis, including O–H (3422 cm⁻^1^), C–H (2931 cm⁻^1^), C = O (1614.9 and 1412 cm⁻^1^), S = O (1251.6 cm⁻^1^), C–O–C glycosidic bonds (1036.9 cm⁻^1^), and α-glycosidic linkages (595.8 cm⁻^1^), consistent with sulfated polysaccharides (Fig. 1D) (Ghanavati et al. 2022; Kong et al. 2021).

**Fig. 2:**
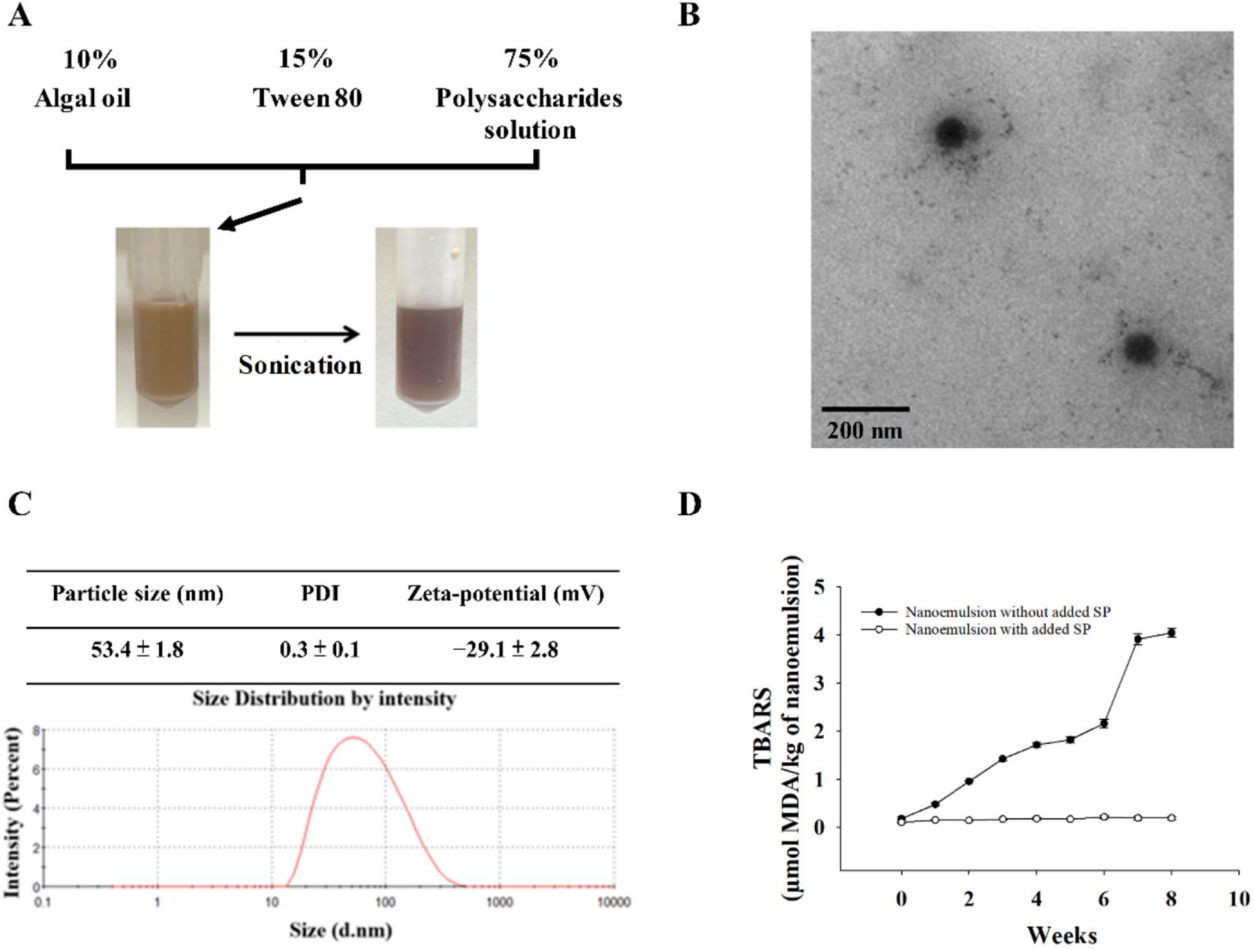
Characterization of SP-AO NE. **A** Diagram of SP-AO NE formulation and preparation process. **B** A representative TEM micrograph illustrating the structure of SP-AO NE. Scale bar = 200 nm. **C** Measurement of particle size, PDI, and zeta potential of SP-AO NE. **D** TBARS values of SP-AO NE and AO NE under 4 °C storage conditions. Data are shown as the mean with standard deviation (n = 3)

Optimization of the NE formulation involved testing various ratios of AO (10–20%), Tween 80 (15–20%), and SP concentration (5–20 mg/mL) in PBS. Results indicated that formulations with over 10% AO or Tween 80 below 15% showed instability, while SP concentrations above 10 mg/mL caused precipitation. The optimal NE was formulated with 10% algal oil, 15% Tween 80, and 75% PBS containing 10 mg/mL SP. Ultrasonication for 20 min produced a clear brown NE, likely due to the inherent color of the SP (Fig. 2A). TEM showed spherical droplets with sizes ranging from 60 to 80 nm (Fig. 2B). DLS measurements yielded an average particle size of 53.4 ± 1.8 nm, a PDI of 0.3 ± 0.1, and a zeta potential of − 29.1 ± 2.8 mV, revealing consistent droplet sizes and reliable electrostatic stability (Fig. 2C). Given the susceptibility of omega-3 polyunsaturated fatty acids in AO to oxidation, TBARS assays were performed to assess lipid oxidation. After 8 weeks at 4 °C, TBARS values in NE without SP increased over 20-fold, whereas NE with SP showed only a twofold increase, demonstrating antioxidative effect of SP on AO NE (Fig. 2D). Additionally, storage stability tests revealed no significant changes in particle size, PDI, and zeta potential at 4 °C, confirming good stability of SP-AO NE. However, at 25 °C and 37 °C, these parameters increased significantly over time, indicating decreased stability. Thus, 4 °C is the preferred storage condition for maintaining NE stability (Table 2).

**Table 2:** Changes in particle size, PDI, and zeta potential of SP-AO NE during 8 weeks of storage at 4 °C, 25 °C and 37 °C

		Week 0	Week 2	Week 4	Week 6	Week 8
4 °C	Particle size (nm)	46.6 ± 0.4^aC^	47.9 ± 1.1^aA^	49.2 ± 1.7^aB^	47.8 ± 1.0^aC^	47.5 ± 0.4^aB^
	PDI	0.3 ± 0.0^aB^	0.3 ± 0.0^aA^	0.3 ± 0.0^aB^	0.3 ± 0.0^aB^	0.3 ± 0.0^aB^
	Zeta-potential (mV)	− 19.2 ± 3.2^aA^	− 16.1 ± 0.8^aA^	− 21.4 ± 2.9^aA^	− 23.5 ± 5.8^aA^	− 21.6 ± 3.5^aC^
25 °C	Particle size (nm)	49.4 ± 0.8^cdB^	48.4 ± 0.5^dA^	51.1 ± 1.3^cAB^	53.8 ± 1.5^bB^	62.6 ± 1.3^aB^
	PDI	0.3 ± 0.0^ dB^	0.3 ± 0.0^dA^	0.3 ± 0.0^cA^	0.4 ± 0.0^bA^	0.4 ± 0.0^aA^
	Zeta-potential (mV)	− 23.6 ± 3.9^bA^	− 14.97 ± 0.5^aA^	− 28.3 ± 5.6^bcA^	− 33.7 ± 7.5^bcA^	− 27.0 ± 2.1^cB^
37 °C	Particle size (nm)	54.6 ± 1.5^bA^	50.0 ± 0.8^bA^	53.9 ± 1.7^bA^	64.2 ± 2.3^bA^	322.9 ± 24.8^aA^
	PDI	0.4 ± 0.0^bA^	0.3 ± 0.0^cA^	0.3 ± 0.0^bcB^	0.3 ± 0.0^bcA^	0.5 ± 0.1^aA^
	Zeta-potential (mV)	− 20.4 ± 1.3^aA^	− 16.1 ± 1.9^aA^	− 21.1 ± 4.5^aA^	− 28.8 ± 7.4^bA^	− 39.9 ± 0.3^cA^

Each value is mean ± standard deviation (n = 3)

^a^^−^^d^Different letters indicate significantly different values (*p* < 0.05) in various storage time with the same temperature

^A^^−^^C^Different letters indicate significantly different values (*p* < 0.05) in various temperature with the same storage time

### Intranasal NE treatment ameliorated IgE-mediated AR

**Fig. 3:**
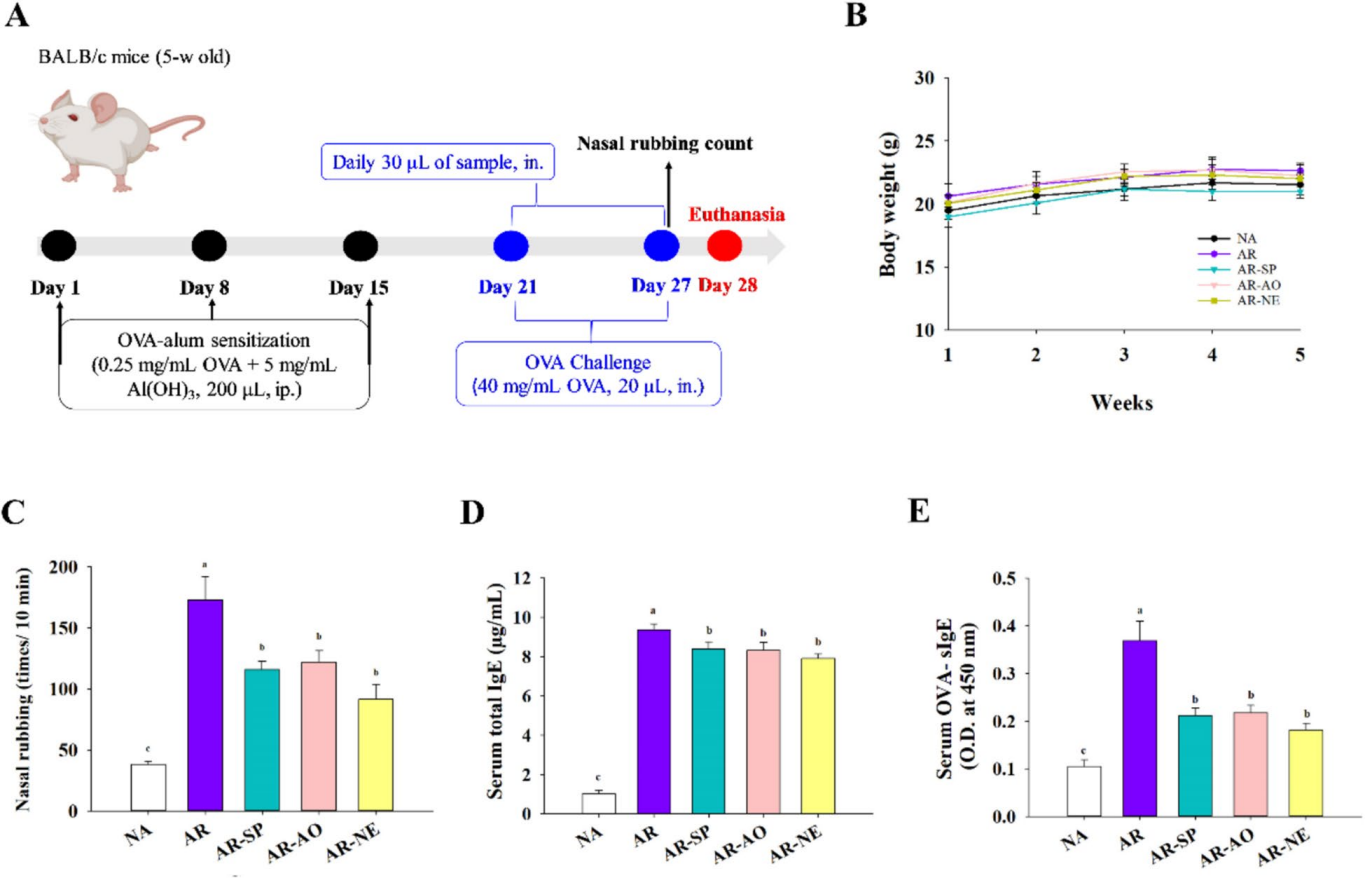
Effect of intranasal SP-AO NE treatment on IgE-mediated AR alleviation. **A** Experimental timeline for AR induction and SP-AO NE treatment. **B** Changes in body weight over the course of the experiment. **C** Frequency of nasal rubbing observed in each group of mice 10 min after the final challenge. **D** total IgE and **E** OVA-sIgE levels in serum. Data are shown as mean ± SEM (n = 6) and reflect one of two independent experiments that consistently demonstrated similar trends. Statistical significance (P < 0.05) is indicated by differing superscript letters (a–c)

Throughout the 28-days experiment (Fig. 3A), mice exhibited steady weight gain, normal behavior, and no visible adverse effects, indicating the treatments were well tolerated (Fig. 3B). Nasal rubbing, a hallmark symptom of AR, was recorded 10 min after OVA challenge on day 27. The AR group showed significantly increased rubbing frequency compared to the naive (NA) controls. Treatment groups receiving SP (AR-SP), AO (AR-AO), or the SP-AO NE (AR-NE) all demonstrated significantly reduced scratching compared to AR mice, indicating symptom relief by the formulations (Fig. 3C). Serum total IgE and OVA-specific IgE (OVA-sIgE) levels were markedly elevated in AR mice relative to NA controls. Both AR-SP and AR-AO groups showed significantly lower total IgE compared to AR mice, with the AR-NE group exhibiting the most pronounced reduction (Fig. 3D). Additionally, OVA-sIgE levels in AR-SP, AR-AO, and AR-NE groups were significantly decreased compared to AR controls (Fig. 3E). These findings suggest that both SP and AO individually modulate allergen-specific immune responses, while their incorporation into a NE further enhances suppression of total IgE production.

### Intranasal NE treatment alleviated histopathological changes in nasal mucosa and lung tissues of AR mice

**Fig. 4:**
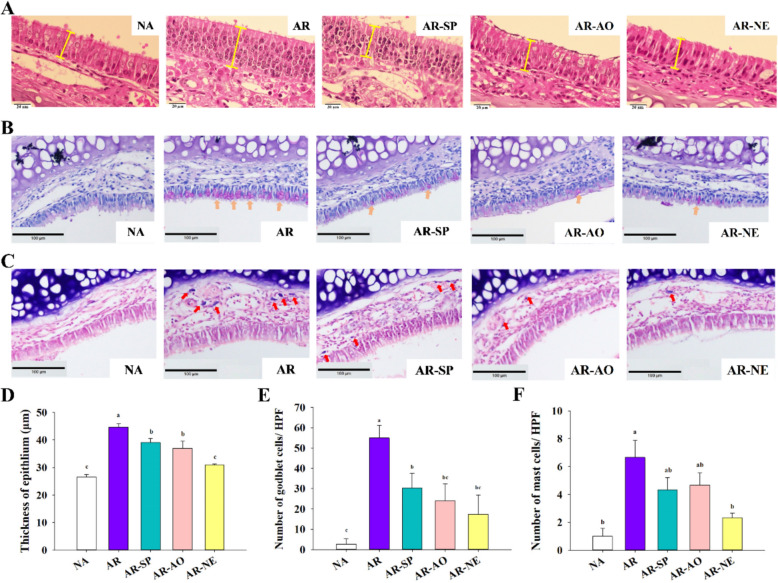
Histopathological changes in nasal mucosa following intranasal SP-AO NE treatment. Illustrative images of nasal tissue after staining with **A** H & E, **B** PAS, and **C** toluidine blue. Magnification: × 1000 for H & E staining, scale bar = 20 μm; × 200 for PAS and toluidine blue, scale bar = 100 μm. The spacing symbols indicate epithelial thickness. The clay-colored arrows point to mucin-producing goblet cells, and the red arrows highlight mast cells. Quantification of **D** epithelial thickness, **E** goblet cell count, and **F** mast cell number was performed using Image J. Data are shown as mean ± SEM (n = 6) and reflect one of two independent experiments that consistently demonstrated similar trends. Statistical significance (*P* < 0.05) is indicated by differing superscript letters (a–c)

AR is characterized by infiltration of inflammatory cells such as mast cells around nasal tissues, alongside goblet cell hyperplasia, leading to mucosal thickening and tissue damage. To evaluate the protective effects of treatments on nasal mucosa, histological analyses were performed using H & E, PAS, and toluidine blue staining. H & E staining at 1000 × magnification showed that the nasal epithelium in the naive (NA) group was intact. In opposition, the AR group exhibited significant epithelial thickening, likely due to inflammation. Treatment with SP (AR-SP), AO (AR-AO), and the NE (AR-NE) all reduced epithelial thickness, with AR-NE showing the most pronounced improvement (Fig. 4A, D). PAS staining demonstrated minimal goblet cells in NA mice, while AR mice displayed marked goblet cell hyperplasia. Treatment groups exhibited notably fewer goblet cells, with AR-NE showing a significant reduction compared to AR controls, indicating effective inhibition of goblet cell proliferation (Fig. 4B, E). Toluidine blue staining revealed mast cell infiltration in the lamina propria of AR mice, significantly higher than in NA mice. Mast cell counts were reduced in AR-SP and AR-AO groups, with AR-NE showing a significant decrease to 2.33 ± 0.33 cells/field (Fig. 4C, F). These results demonstrate that intranasal SP–AO NE treatment effectively suppresses cell infiltration and goblet cell hyperplasia within the nasal mucosa, alleviating histopathological damage associated with AR.

**Fig. 5:**
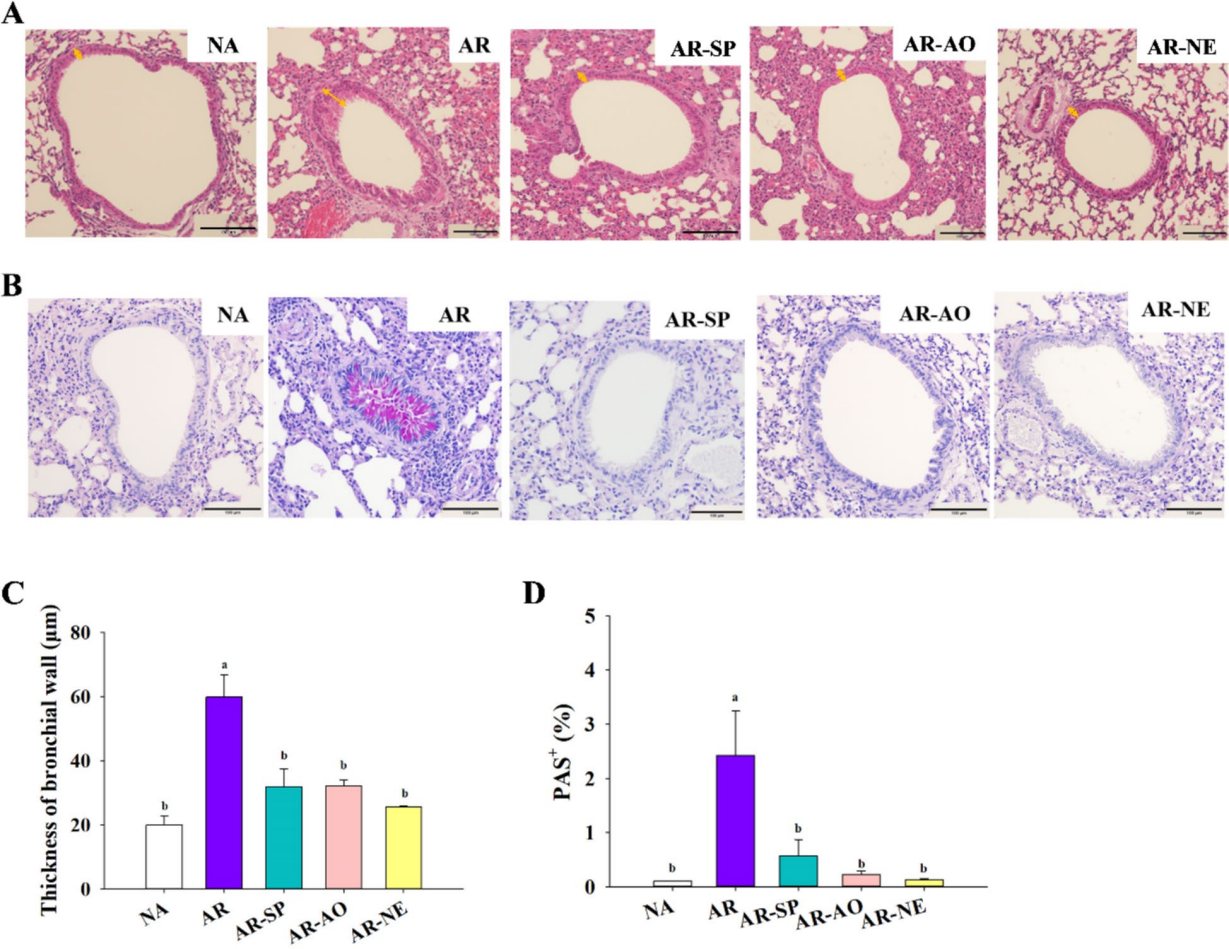
Histopathological changes in lung tissue following intranasal SP-AO NE treatment. Illustrative images of lung tissue after staining with **A** H & E and **B** PAS. Magnification: × 200, scale bar = 100 μm. Bidirectional arrows denote the thickness of the bronchial wall. Purple staining indicates bronchial mucin. Quantification of **C** bronchial wall thickness and **D** percentage of PAS^+^ area was conducted using Image J. Data are shown as mean ± SEM (n = 6) and reflect one of two independent experiments that consistently demonstrated similar trends. Statistical significance (*P* < 0.05) is indicated by differing superscript letters (a–b)

Considering that nasal inflammation may affect the lower respiratory tract, lung tissues from all groups were further examined for pathological changes. H & E staining revealed significant bronchial wall thickening and extensive inflammatory cell infiltration in the AR group. Although the AR-SP and AR-AO groups showed slight reductions in bronchial wall thickness, inflammatory cell infiltration was still evident. In contrast, the AR-NE group demonstrated marked improvements in both bronchial wall thickness and inflammatory cell infiltration (Fig. 5A, C). An elevated number of goblet cells of AR mice was detected using PAS staining, which was noticeably reduced in the AR-SP, AR-AO, and AR-NE groups (Fig. 5B). Quantitative analysis of PAS-positive areas confirmed significantly higher goblet cell proliferation in AR mice compared to NA controls, with all treatment groups showing significant reductions relative to AR (Fig. 5D). Overall, these findings suggest that AR induces bronchial wall thickening and inflammatory infiltration, while intranasal administration of the SP-AO NE effectively mitigates lung tissue pathological damage.

### Intranasal NE treatment modulated production of antibodies and cytokines

**Fig. 6:**
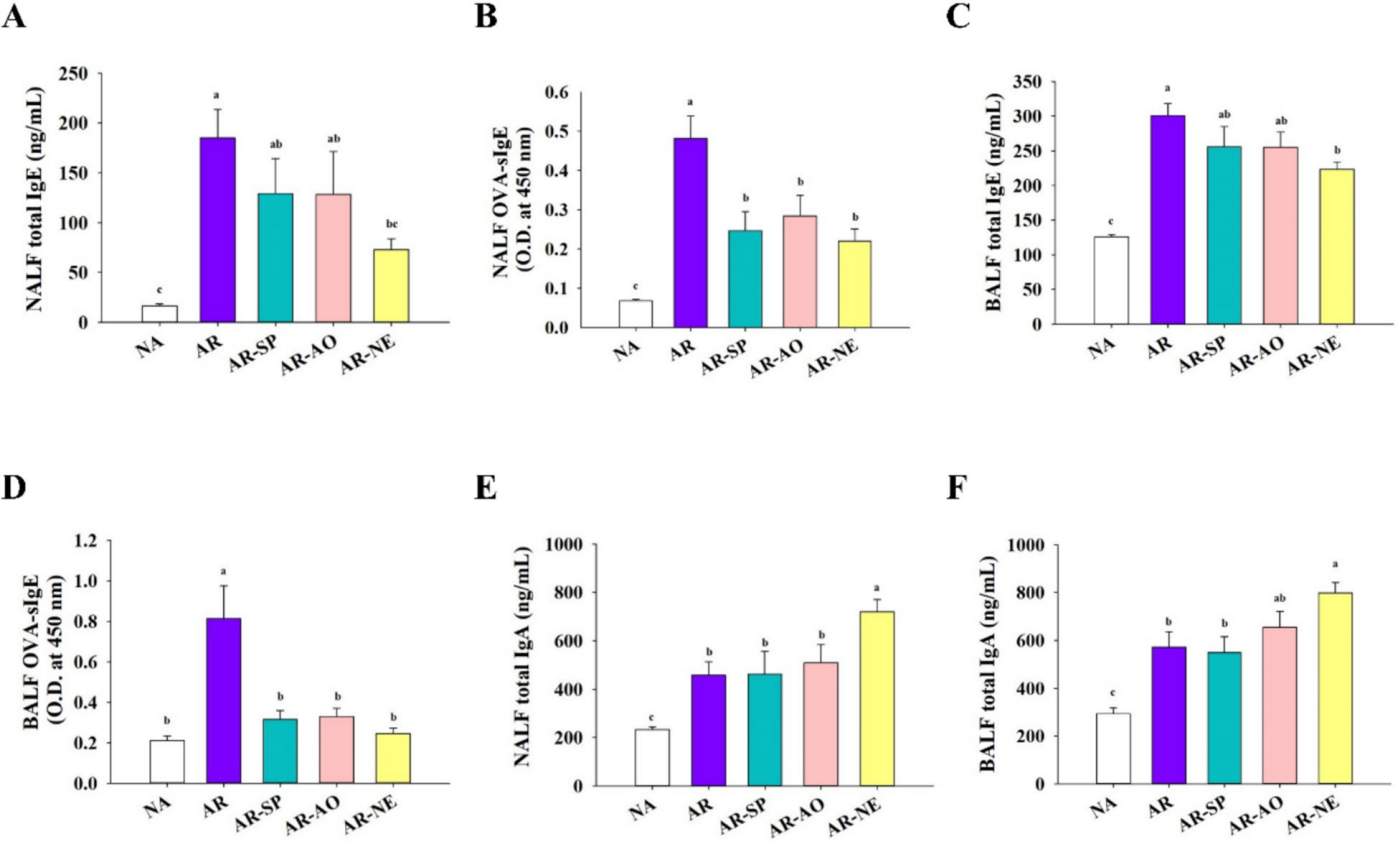
Modulation of nasal antibody production by intranasal SP-AO NE treatment. The levels of **A** total IgE, **B** OVA-sIgE in NALF, **C** total IgE, **D** OVA-sIgE in BALF, and **E**, **F** total IgA in both NALF and BALF were measured by ELISA. Data are shown as mean ± SEM (n = 6) and reflect one of two independent experiments that consistently demonstrated similar trends. Statistical significance (*P* < 0.05) is indicated by differing superscript letters (a–c)

Following OVA induction, AR mice showed significantly elevated total IgE and OVA-sIgE levels in NALF and BALF compared to the NA group. Total IgE levels in the NALF and BALF of mice treated with AR-SP or AR-AO were comparable to those in the AR group. Notably, the AR-NE treatment resulted in a significant decline in IgE concentrations in both fluids relative to the AR group (Fig. 6A, C). All treated groups exhibited a notable reduction in OVA-sIgE levels in NALF and BALF relative to the AR group (Fig. 6B, D). These results indicate that both SP and AO can modulate allergen-specific immune responses, while the SP-AO NE formulation further enhances the regulation of total IgE production in the airway. IgA is the most abundant immunoglobulin on mucosal surfaces, serving an essential function in immune regulation and the first line of humoral defense against mucosal antigens (El Ansari et al. 2025). The impact of treatments on mucosal immune function was evaluated by measuring total IgA in NALF and BALF. Compared to the NA group, all OVA-induced groups showed significantly increased total IgA and OVA-sIgA expression. While AR-SP and AR-AO had little effect on total IgA compared with AR, AR-NE significantly enhanced IgA levels in both NALF and BALF compared to AR (Fig. 6E, F). These results demonstrate that intranasal administration of the SP-AO NE notably enhances mucosal total IgA production, potentially contributing to improved mucosal immunity in AR.

**Fig. 7:**
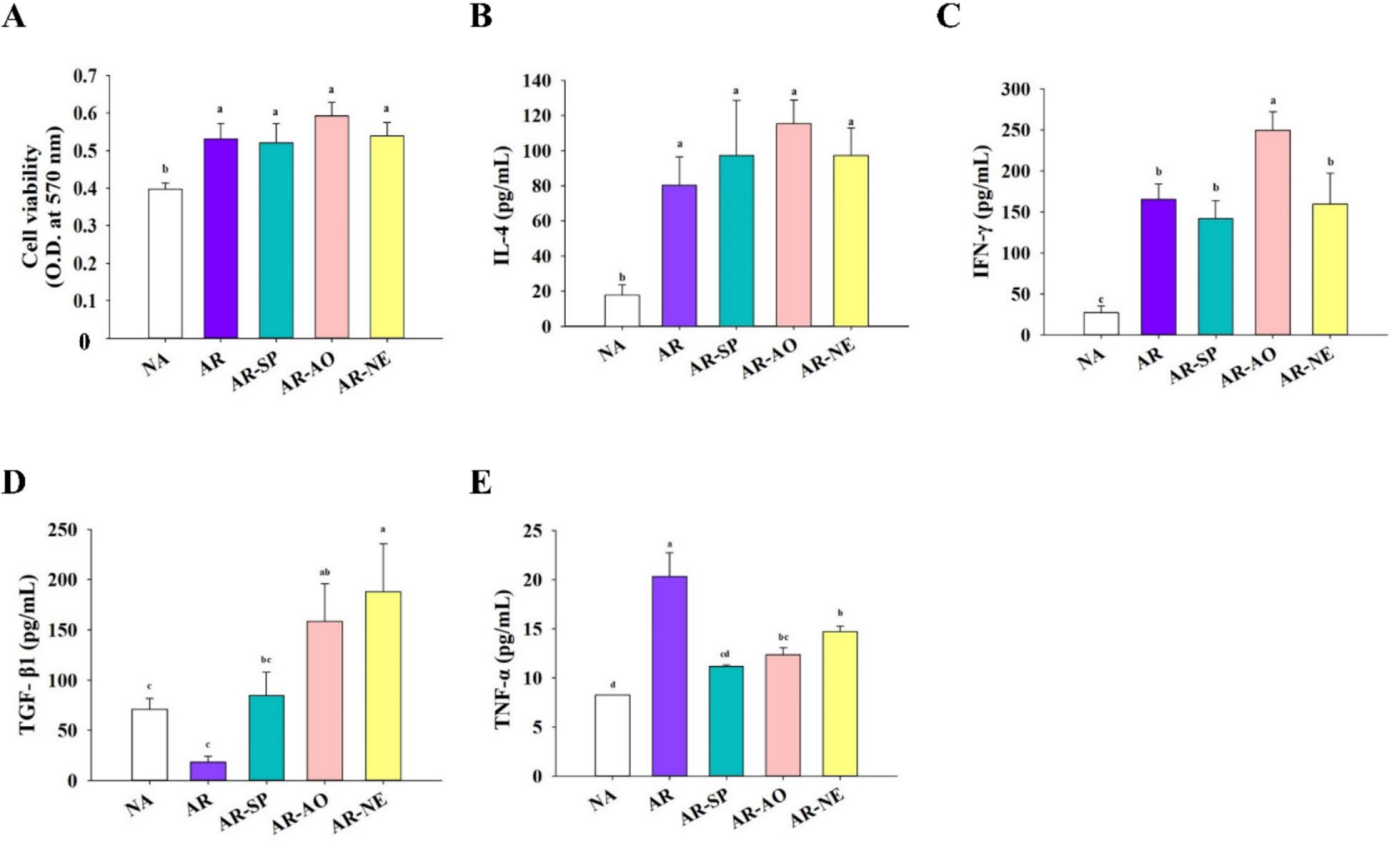
Intranasal SP-AO NE treatment impacts splenic cytokine production. Following euthanasia, spleens were isolated for splenocyte preparation. Splenocytes were incubated with OVA for 48 h, and **A** cell viability, and concentrations of **B** IL-4, **C** IFN-γ, **D** TGF-β1, and **E** TNF-α in the supernatants were measured. Data are shown as mean ± SEM (n = 6) and reflect one of two independent experiments that consistently demonstrated similar trends. Statistical significance (*P* < 0.05) is indicated by differing superscript letters (a–c)

To evaluate the systemic immune response, spleens were harvested from sacrificed mice, and splenocyte suspensions were prepared. After 48 h of culture, splenocyte viability was assessed by MTT assay. Splenocyte activity in AR mice was significantly elevated. However, the viability of splenocytes remained comparable across all treatment groups and the AR control (Fig. 7A). To investigate the Th1/Th2 balance and immune regulation, cytokine secretion in OVA-stimulated splenocyte culture supernatants was measured. The AR group showed significantly increased expression of IL-4 and IFN-γ compared to NA controls (Fig. 7B, C). Treatment with the samples did not significantly alter IL-4 levels relative to the AR group (Fig. 7B). Notably, a notable enhancement in IFN-γ expression was observed in the AR-AO group versus the AR control, whereas AR-SP and AR-NE groups showed no significant difference in IFN-γ expression relative to AR (Fig. 7C). Treg cells regulate immune tolerance and suppress inflammatory cells such as mast cells through secretion of TGF-β1 (Conrad et al. 2025). Both AR-AO and AR-NE groups exhibited significantly higher TGF-β1 levels than the AR group (Fig. 7D). Furthermore, AR induction led to a significant rise in the pro-inflammatory cytokine TNF-α relative to NA mice, while AR-NE treatment effectively suppressed TNF-α expression (Fig. 7E).

**Fig. 8:**
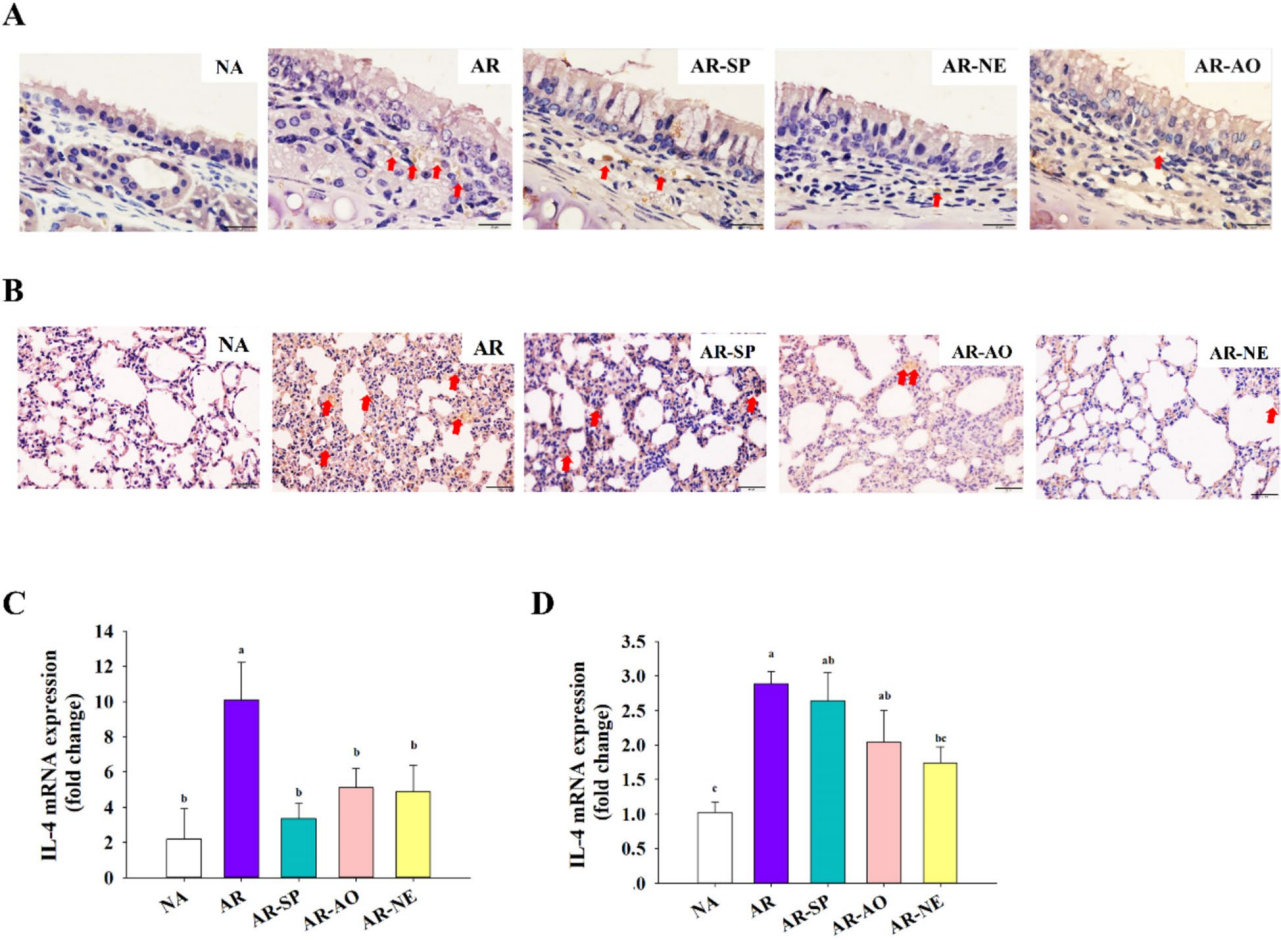
Effects of intranasal SP-AO NE on airway IL-4 expression. Representative images of IL-4 IHC staining in **A** nasal mucosa and **B** lung tissues. Magnification: × 1000. Red arrows indicate positive IL-4 staining (brown). IL-4 mRNA expression in **C** nasal mucosa and **D** lung tissues was assessed by RT-qPCR. Data are shown as mean ± SEM (n = 6) and reflect one of two independent experiments that consistently demonstrated similar trends. Statistical significance (*P* < 0.05) is indicated by differing superscript letters (a–c)

To assess how the treatment modulated Th2-mediated immunity in the nasal and pulmonary tissues, IHC staining and RT-qPCR analyses were performed to assess IL-4 expression in nasal and lung tissues from all groups. IHC results indicated negligible IL-4 in NA nasal tissues compared to the robust expression seen in AR mice. Compared to the AR group, IL-4 expression was reduced in the AR-SP, AR-AO, and AR-NE groups (Fig. 8A). Similarly, in lung tissues, IHC results indicated that AR induced high IL-4 expression, which was attenuated in the AR-NE-treated group (Fig. 8B). Consistently, RT-qPCR assessment revealed substantially elevated IL-4 mRNA in the nasal mucosa of AR mice versus NA controls. Notably, all treatment groups exhibited a notable reduction in nasal IL-4 gene expression relative to AR (Fig. 8C). In parallel, IL-4 mRNA levels in lung tissues were significantly elevated in AR mice relative to NA controls. Lung IL-4 gene expression was notably suppressed in the AR-NE group versus AR (Fig. 8D). These findings indicate that the SP-AO NE effectively downregulates IL-4 expression, suggesting an inhibitory effect on Th2 immune responses in the respiratory tract.

## Discussion

Polysaccharides are critical bioactive components of *Sargassum*, constituting 20–70% of its dry weight (Chang et al. 2023). SP has been widely reported to exhibit anti-inflammatory and immunoregulatory properties, with higher sulfate content often associated with enhanced antioxidant activity (Chen et al. 2021). The molecular mass of SP in this study ranged from 874.4 to 1.2 kDa, with fucose, glucose, and galactose being the dominant monosaccharides. These characteristics are consistent with previous reports describing comparable monosaccharide composition and molecular weight ranges for SP (Kong et al. 2021; Chen et al. 2020). The incorporation of SP into a NE system enables improved stability and bioavailability, key factors for efficient bioactive delivery. The resulting SP–AO NE exhibited a narrow droplet size distribution (20–200 nm), with an average diameter of 53.4 ± 1.8 nm and a low polydispersity index (0.3 ± 0.1), indicative of good homogeneity. These properties are known to contribute to improved transparency and physical stability in NE systems (Choi and McClements 2020; Che Marzuki et al. 2019). TEM further confirmed the spherical morphology and uniform size distribution of the droplets, supporting successful formulation.

The SP–AO NE retained physicochemical stability at 4 °C for 8 weeks, but showed decreased stability at 25 °C and 37 °C, likely due to increased molecular movement and droplet collision (Shi et al. 2022). Given the high polyunsaturation of DHA, which is prone to oxidation, these results are consistent with expectations. Our findings suggest that incorporation into the NE, together with SP, may reduce lipid oxidation, supporting enhanced DHA stability during storage, as observed in other NE-based systems (Zhang et al. 2019a; Karthik and Anandharamakrishnan 2016). This aligns with previous studies showing that sulfated polysaccharides can act as free radical scavengers and improve the stability of omega-3 fatty acids (Kang et al. 2019; Salvia-Trujillo et al. 2016).

In the employed AR model, intranasal administration of SP–AO NE alleviated clinical and histopathological features, including nasal rubbing, epithelial thickening, and increased goblet and mast cell counts. The effects of SP or AO alone were less pronounced, suggesting additive or synergistic benefits of the combined NE formulation. This observation is consistent with the role of mast cells in AR, where their activation releases inflammatory mediators (Piao et al. 2019). Reduced mast cell infiltration may reflect improved local delivery and retention of bioactive compounds in the nasal mucosa. Histopathological analysis of lung tissues revealed attenuation of AR-associated lung pathology, including bronchial wall thickening and inflammatory cell infiltration. These findings suggest that SP–AO NE may influence immune interactions between the nose and lungs, supporting the concept of a nasal–pulmonary reflex in AR (Liu et al. 2022).

Immune-related effects of SP–AO NE were evaluated by measuring total IgE and OVA-sIgE levels. While SP or AO alone did not significantly reduce IgE, SP–AO NE decreased both total and allergen-specific IgE compared with the untreated model group. These findings suggest an association with attenuation of allergen-driven responses in this AR model, although they do not establish direct modulation of specific immune cell subsets or signaling pathways. SP–AO NE also increased IgA levels in NALF and BALF, which may reflect enhanced mucosal immune status. In addition, TGF-β1 was upregulated and TNF-α was reduced in NE-treated mice, consistent with a shift toward a less inflammatory profile (Conrad et al. 2025). However, these observations remain associative, and potential involvement of Treg-related mechanisms is inferred rather than directly demonstrated. Local IL-4 expression, a cytokine known to promote IgE production and contribute to epithelial barrier dysfunction in AR (Jung et al. 2023), was decreased following SP–AO NE treatment. This reduction may be related to improved mucosal conditions and partial mitigation of inflammatory responses.

The therapeutic effects of SP–AO NE were comparable to prior studies using higher doses of *Sargassum* extracts or omega-3 fatty acids. For example, intraperitoneal administration of *Sargassum* extracts (300 and 600 mg/kg) has been shown to reduce AR symptoms and inflammatory markers (Zhang et al. 2019b). Similarly, omega-3 fatty acids (8% in the diet) have been reported to reduce mast cell degranulation and inflammation in AR models (Tabaru et al. 2024). Unlike these systemic or oral approaches, this study reports, to our knowledge, the first NE-based intranasal co-delivery system combining SP and AO for AR. The novelty lies in the formulation strategy and localized delivery rather than in new bioactive discovery. The intranasal route enhances local delivery and bypasses hepatic first-pass metabolism, allowing more direct action at the site of inflammation (Chaturvedi et al. 2011).

The SP–AO NE demonstrated promising therapeutic effects in a murine AR model, providing insights into local immune modulation and treatment efficacy. NE particles < 200 nm have been reported to efficiently penetrate nasal barriers and remain long enough to influence mucosal immune responses (Huang et al. 2020). Although cytokine profiling and histopathology support anti-inflammatory and immunomodulatory effects, the precise molecular mechanisms remain to be elucidated. Therefore, future studies are warranted to substantiate these mechanistic hypotheses, including quantitative evaluation of nasal deposition and cellular uptake, detailed immune cell phenotyping, gene expression analyses, and pathway-specific validation of key signaling axes. This study focused on evaluating the therapeutic potential of intranasal SP-AO NE, and to minimize animal use, positive controls and dose–response analyses were not included. These will be addressed in future studies alongside comparisons with clinically approved intranasal therapies to strengthen mechanistic and clinical relevance. Although comprehensive in vivo safety assessments were not performed, animals showed no behavioral or physiological abnormalities, aside from transient nasal rubbing, and body weight remained stable, indicating that SP-AO NE was well tolerated under the tested conditions. Further work is needed to assess long-term safety, formulation stability, and clinical translatability.

## Conclusion

The SP–AO NE developed in this study represents a promising intranasal treatment for managing AR. The formulation demonstrated good physicochemical stability, effectively protected AO from oxidation, and provided therapeutic benefits in a mouse model of AR. It modulated immune responses, including suppression of IgE, enhancement of mucosal IgA, and regulation of cytokines such as TGF-β1 and TNF-α, while supporting nasal epithelial integrity. These findings suggest that SP–AO NE is a biocompatible system capable of localized delivery of algal-derived bioactives and may offer a natural strategy for mitigating allergic airway inflammation.

## Data Availability

Data will be made available on request.

## Abbreviations

AO: Algal oil
AR: Allergic rhinitis
BALF: Bronchoalveolar lavage fluid
DHA: Docosahexaenoic acid
DLS: Dynamic light scattering
ELISA: Enzyme-linked immunosorbent assay
FTIR: Fourier-transform infrared spectroscopy
HPLC: High-performance liquid chromatography
H & E: Hematoxylin–eosin
IFN: Interferon
Ig: Immunoglobulin
IHC: Immunohistochemical
IL: Interleukin
MTT: 3-(4,5- Dimethylthiazol-2-yl)-2,5-diphenyltetrazolium bromide
NA: Naive NALFnasal lavage fluid
NE: Nanoemulsion
OVA: Ovalbumin
PAS: Periodic acid–Schiff
PBS: Phosphate-buffered saline
PDI: Polydispersity index
RT-qPCR: Reverse transcription quantitative polymerase chain reaction
sIg: Specific Ig
SP: *Sargassum* Polysaccharides
TBARS: Thiobarbituric acid reactive substances
TEM: Transmission electron microscope
Th: T helper
TGF: Transforming growth factor
Treg: Regulatory T
